# Understanding the social worker–family relationship through self‐determination theory: A realist synthesis of Signs of Safety

**DOI:** 10.1111/cfs.12903

**Published:** 2022-01-26

**Authors:** Louise Caffrey, Freda Browne

**Affiliations:** ^1^ Social Policy, School of Social Work & Social Policy Trinity College Dublin Dublin Ireland; ^2^ General Nursing, School of Nursing, Midwifery and Health Systems University College Dublin Dublin Ireland

**Keywords:** child protection, realist synthesis, self‐determination theory, signs of safety, social work

## Abstract

Signs of Safety (SofS) is a popular framework for child protection social work practice, used in more than 200 jurisdictions worldwide. Although workers tend to find SofS tools easy to use, skilled application of the approach is challenging, and research has found that SofS is often not implemented as intended. This study aimed to deepen and inform the explanation (initial theory) of what key SofS tools and processes are expected to achieve in the family–worker interaction and why. A realist synthesis was used, involving a realist review of literature and focus groups with 22 international SofS experts. Using self‐determination theory, we detail how SofS can be conceptualized as aiming to support families to experience ‘autonomous’ rather than ‘controlled’ motivation by supporting basic human needs for ‘autonomy’ (feeling a sense of volition), ‘competence’ (feeling effective) and ‘relatedness’ (feeling cared for). This explanation can be used for training and evaluation purposes to better explain and test how SofS expects to engage families in the child protection process. More broadly, we suggest that self‐determination theory may contribute a mechanistic explanation of effective social work practice more generally and that this hypothesis should be empirically investigated.

## 1 INTRODUCTION

Signs of Safety (SofS) is a widely used framework for child protection social work and has been implemented in approximately 200 jurisdictions globally (Turnell & Murphy, 2017). It is used in more than half of local authorities in England (Baginsky et al., 2020) and is the national practice framework in the Republic and Northern Ireland. A defining feature is that SofS aims to provide families and the individuals naturally connected to them with a genuine opportunity to demonstrate that they can provide safe care for their children. The approach builds on the participatory approaches of solution‐focused (brief) therapy and strengths‐based practice, aiming to help professionals build constructive helping relationships while maintaining a strong focus on safety and rigorous exercise of professionals' statutory role. Heavy emphasis is placed on working with families and children to develop risk assessments and safety plans and on building constructive relationships between professionals themselves. Further, it aims to support professionals to reach balanced conclusions by incorporating an analysis of strengths alongside danger and harm and encouraging professionals to approach the assessment with an open mind (Turnell & Murphy, 2017).

The current SofS theory of change sets out a detailed description and illustration of each step that is expected to happen in the SofS causal pathway, and it further outlines how those changes are influenced by the organizational context SofS is introduced into (Munro et al., 2016). However, previous research has found that SofS is often implemented in a piecemeal or incomplete fashion and that it is sometimes misinterpreted and implemented incorrectly (Baginsky et al., 2017; Baginsky et al., 2021; Rijbroek et al., 2017; Roberts et al., 2019; Rothe et al., 2013). In one organization ostensibly using SofS, observations of practice found ‘there were many visits when not a single identified element of SofS was used in [social workers'] interaction with families' (Baginsky et al., 2021, p. 28). Indeed, the literature suggests that although SofS tools can be easily used, overall workers find SofS challenging to apply in the field, skilled use of the approach requires time (Baginsky et al., 2021; Skrypek et al., 2012) and there may be confusion amongst some workers about ‘what SofS is and how to use it’ (Baginsky et al., 2021, p. 10).

In a recent mixed methods systematic review, incorporating a realist synthesis, Sheehan et al. (2018) explored the underlying programme theory of SofS from the existing literature. Our paper does not intend to replicate this work but to deepen and theoretically inform the explanation (initial theory) of a key aspect of the SofS approach: How and why social workers' use of SofS tools and processes is thought to offer opportunities to support family members' motivation to engage in the child protection process and thus maximize the family's potential to build safety around their child. In this paper, we combine existing SofS literature with self‐determination theory (SDT) and expert focus groups to provide a deeper, theoretically informed explanation of what key SofS tools and processes are expected to achieve in the family–worker interaction and why. At an overarching level, this paper will detail how SofS can be conceptualized as aiming to support families to experience ‘autonomous’ rather than ‘controlled’ motivation by supporting basic human needs for ‘autonomy’ (feeling a sense of volition), ‘competence’ (feeling effective) and ‘relatedness’ (feeling cared for). The detailed explanation we present in this paper can be used for training purposes to better explain how and why SofS is expected to engage and motivate families in the child protection process and for the purpose of evaluation to test and develop this theory of change.

## 2 SELF‐DETERMINATION THEORY

SDT is a psychological theory of behaviour change that is supported by a strong evidence base across a wide array of fields including health care, education, work, sport and psychotherapy (Ryan & Deci, 2017). The theory is interested in how people may internalize and integrate behaviours that are externally motivated so that they themselves regulate and engage in these behaviours sustainably.

In the field of psychology, motivation is typically differentiated between that which is ‘extrinsically’ motivated (motivated by an external push) and ‘intrinsically’ motivated (self‐motivated). However, SDT offers another category, suggesting that motivation that is ‘autonomous’—that is, engaged in while feeling some element of voluntariness or willingness—is more effective than motivation that is ‘controlled’ (i.e., feeling coercive pressure to engage in it) for promoting people's behaviour change, satisfaction and well‐being (Ryan & Deci, 2017).

SDT suggests that under the right circumstances, it is possible for people to internalize behaviours that are extrinsically motivated so that they come to personally value them, feel ownership of them, accept and choose them. SDT has demonstrated that people tend to experience ‘autonomous motivation’ when three basic human needs are satisfied. The basic needs are for *autonomy* (feeling of voluntariness, of being the origin on one's own behaviours, not to be confused with independence), *competence* (feeling effective) and *relatedness* (feeling social connection to others, brought about by feeling understood and cared for by others or through caring for others) (Ryan & Deci, 2017). Summing up the large body of SDT empirical research, Ryan and Deci (2017, p. 9) state

When individuals experience need‐thwarting environments, such as contexts that are overly controlling, rejecting, critical and negative or that otherwise frustrate autonomy, relatedness and competence needs, individuals are more likely to become self‐focused, defensive, amotivated, aggressive, and antisocial […]

A large body of evidence suggests that ‘autonomous motivation’ is associated with more effective and longer lasting behaviour change and greater client wellness, compared with standard care. These findings hold across randomized control trials of, for example, tobacco use, physical activity, weight loss, medication adherence and dental self‐care (Ryan & Deci, 2017, pp. 459–460), health care and health promotion (Ng et al., 2012).

To the best of our knowledge, SDT has not been tested in the context of child protection social work practice. However, van der Helm and colleagues (2018) recently used an SDT framework, demonstrating that an open group climate with low levels of institutional oppression was associated with treatment motivation in adolescents residing in involuntary residential care. While the mandated context of child protection clearly presents an important nuance, mandated referral is not incompatible with SDT because, as noted above, the ‘autonomy’ referred to in SDT is not the same as independence. Rather, it is about helping individuals recognize that they can make choices regarding their behaviours such that behaviours become self‐endorsed, feel in keeping with the person's own interests and values and are engaged in willingly (Ryan & Deci, 2017).

## 3 METHODS

This study employed a realist synthesis (Pawson et al., 2005). Realist methods are well established, particularly within health sciences research (Moore et al., 2015), but thus far have seen little uptake in social work despite calls to embrace the approach (Kazi, 2003). Realist synthesis is primarily a literature reviewing methodology in which a wide array of literature, from peer‐reviewed research studies to grey literature and programme guidance documentation, are used. Realist synthesis aims to produce and refine a deep understanding of how the programme (here, Signs of Safety) works, for whom, in what contexts and why. The approach therefore represents a major departure from conventional systematic reviews in that its goal is explanatory rather than summative (Pawson, 2013).

The concept of mechanism is central to the approach and to this paper, which reports on the development of a key aspect of the programme theory, to explore what key SofS tools and processes are expected to achieve in the family–worker interaction and why. Mechanisms refer to the deeper, often unobservable, ‘underlying causal processes’ that generate behaviour and so inform outcomes (Westhorp, 2014). They are usually found in the cognitive or emotional reasoning responses of stakeholders to resources (material, social or cognitive) offered by programmes and are triggered (or not) in contexts (Pawson & Tilley, 1997).

In a realist synthesis, nuggets of theories about how the programme works may come from the literature on SofS as well as from diverse fields outside of child welfare. Literature is purposively selected based on its potential to contribute to the theory of how and why the intervention works, rather than on its methodological credentials (Pawson, 2013). Realist synthesis then seeks to use the literature and stakeholder focus groups to support, refute or refine the programme theory. Through ‘retroduction’, realist methods aim to produce deeper explanations by seeking to theorize what each programme strategy *does*, or combinations of strategies *do*, in varying contexts (Jagosh, 2020), The process involves imagination, creativity and innovation alongside scientific method (Mingers, 2014) and success depends, in part, on finding concepts at the right level of abstraction or generality (Cartwright & Hardie, 2012). In keeping with the approach of Greenhalgh et al. (2016), we focused on developing the theory of what key SofS tools and processes are expected to achieve in the family–worker interaction and why. We offer them as emerging theories that can be used to guide implementation and it would be helpful if future research explicitly tested and developed them.

**FIGURE 1:**
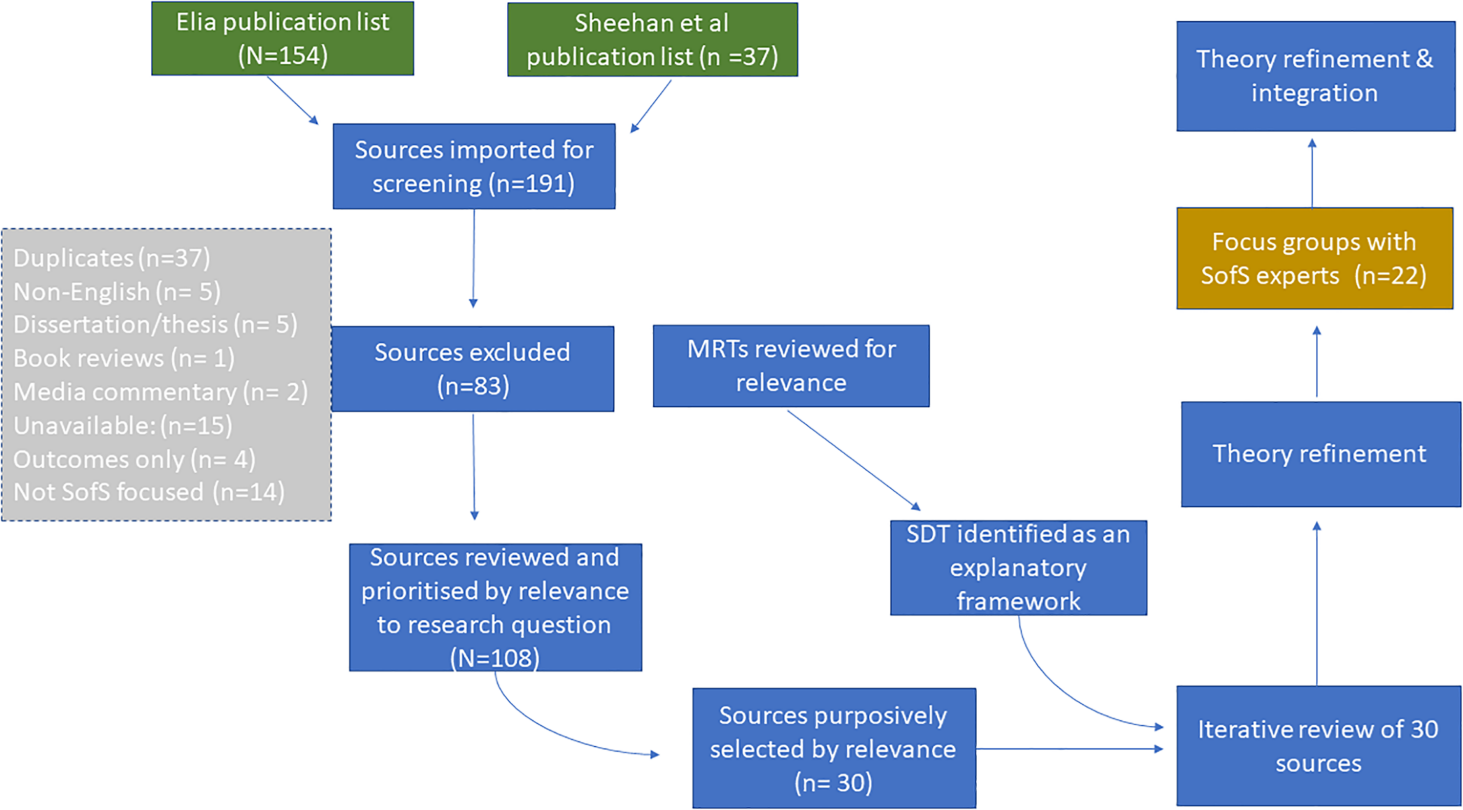
The methodological process

In this study we reviewed three literature sources (a) programme documents created by the SofS programme architects (b) the SofS empirical literature and (c) wider theory outside the area of child welfare that might hold explanatory potential for SofS. Rather than recreating a search for literature we built on the work of colleagues, using the 2019 Elia^1^ list of SofS publications and Sheehan et al. (2018) bibliographical list from their mixed methods systematic review of SofS. While Sheehan and colleagues sought empirical research, the Elia list includes a wider array of sources that mention SofS. Figure 1 outlines the methodological process.

Following application of exclusion criteria, the abstract or introduction for 108 publication was examined and, using an appraisal form, the authors together agreed which documents to purposively include in the review, prioritizing those with most information relevant to developing the theory of what SofS tools and processes are expected to achieve in the family–worker interaction and why. A final set of 30 documents was agreed by the authors. To deepen the explanation, we sought ‘Middle Range Theory’ (MRT) (Merton, 1949) of potential relevance to SofS. In line with the retroductive process, we experienced identifying candidate MRTs as a creative process. To identify MRT, the first author engaged in an iterative process of mentally moving up and down a ‘ladder of abstraction’ (Cartwright & Hardie, 2012, p. 79) with concrete SofS strategies situated at the bottom and the search for more abstract, general theory situated above, constantly asking, ‘what does the cause do?’ (Cartwright, 2020) and ‘what is this strategy, and SofS in general, an example of?’ (Shearn et al., 2017). The following candidate theories were reviewed, social learning theory (Bandura, 1977), diffusion of innovation theory (Rogers, 2003), normalization process theory (May & Finch, 2009), complexity theory (Stacey, 2011) and the working alliance (Horvath & Leslie, 1994). SDT was selected as it held strong explanatory potential to logically root and link the mechanisms emerging from the review of the SofS literature in mechanisms derived from a more abstract theory. For example, our review identified feelings of choice, responsibility and an element of control as important mechanisms in SofS. SDT's conceptualization of ‘autonomous motivation’ seemed to provide an overarching, well‐developed mechanism to group these lower‐level causal mechanisms in terms of Cartwright's (2020) question, ‘what does the cause do?’. Moreover, we found that SofS strategies mapped strikingly well onto those SDT research has found generally support autonomous motivation and the three basic human needs. In keeping with Jagosh et al.'s experience (2012), SDT was not identified and selected as the theoretical framework until after the literature appraisal and review process was underway. We therefore iteratively re‐reviewed the selected literature following identification of SDT.

To scrutinize and develop our findings from the review of the literature, the first author held four focus groups totalling 22 SofS experts. Participants were grouped by their role, comprising SofS architects, Elia directors and two groups with SofS consultants and trainers. Participants were from nine countries across four continents. Focus groups were held on a secure online platform, audio and video recorded and transcribed verbatim. In realist qualitative inquiry, data collection is used to test and refine initial theories developed through reading the literature. Therefore, participants are presented with the rough initial theories, built around SDT, and asked to comment on them given their real‐world experiences (Manzano, 2016). Data analysis was managed using NVivo 12 software and analysed thematically focusing on realist concerns of context, mechanism, outcome configurations within an SDT theoretical framework. The study was approved by the School of Social Work and Social Policy Research Ethics Committee at Trinity College Dublin. All participants provided written informed consent to participate and to be named. Participants' specific contributions are anonymized, but a list of participants is provided in Appendix A.

## 4 FINDINGS

### 4.1 Overarching theory: ‘Autonomously motivated’ behaviour change

Although no connection is made to SDT in SofS's current theory of change, our review suggests that SDT can provide a coherent theoretical framework to explain a key element of SofS: How SofS tools and processes are expected to support the family's motivation to engage in the child protection process. SofS asserts that motivation to change is an attribute, not simply of families, but of the interaction between families and workers, and is not static, but influenced by time and context. The aim should be ‘to create a context that maximizes the likelihood of family members displaying their motivation’ and in doing so, maximize the family's potential to build safety around their child (Turnell & Edwards, 1999, p. 41).

**FIGURE 2:**
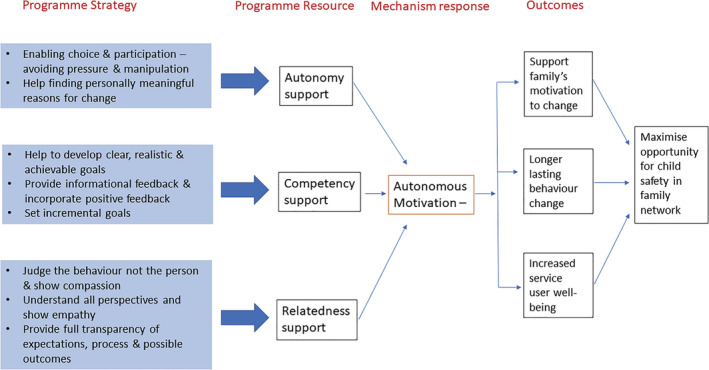
Signs of Safety's theory of change underpinned by self‐determination theory

This keystone of the SofS programme is in direct alignment with SDT's empirically supported claim that clients' motivation is not static but can be either supported or thwarted depending on how well the environment supports basic psychological human needs. Indeed, SDT research documents that in social contexts where basic needs are satisfied, people's motivation and well‐being are more likely to be demonstrated leading to longer lasting behaviour change because individuals internalize and integrate the motivation to engage (Ryan & Deci, 2017). In what follows, we demonstrate that SofS's strategies mirror those that SDT research has found can create a supportive environment for change by satisfying the human need for ‘autonomy’, ‘competence’ and ‘relatedness’. Figure 2 provides an overview of the theoretical model, integrating SDT into the explanation of how SofS can be expected to achieve its aims. In the sections that follow we provide a detailed narrative account.

**FIGURE 3:**
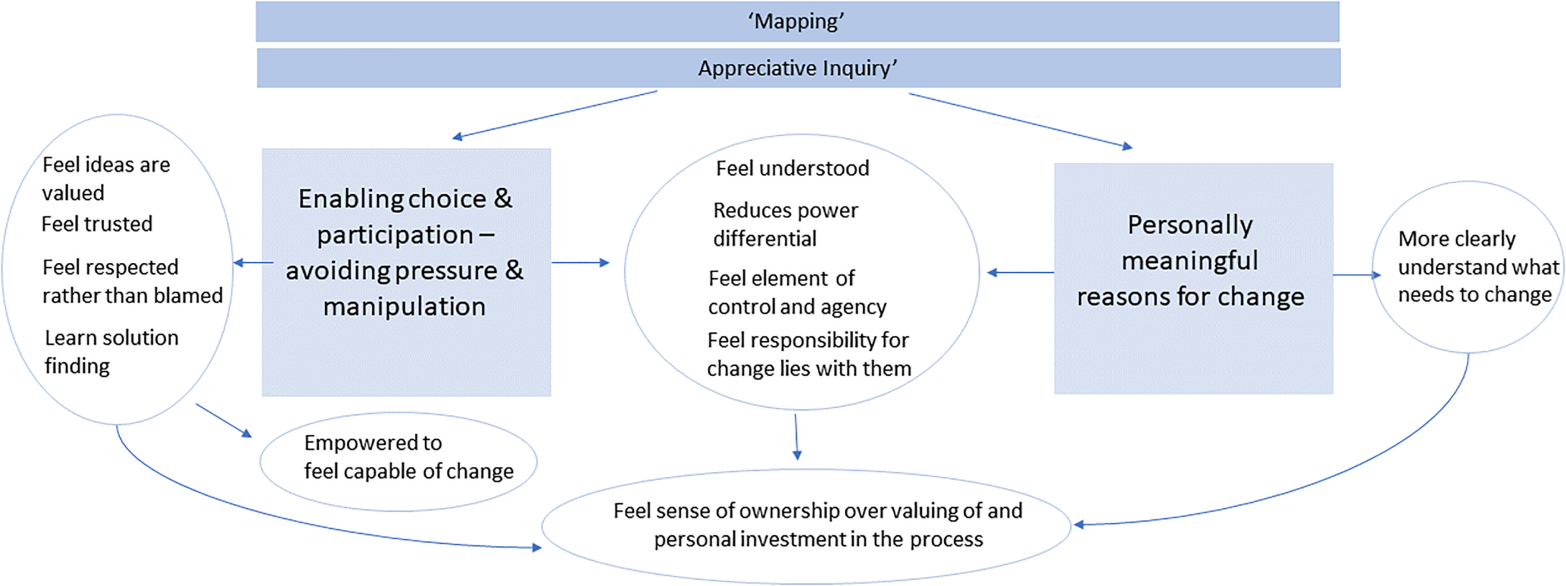
Autonomy supporting strategies and mechanisms

**FIGURE 4:**
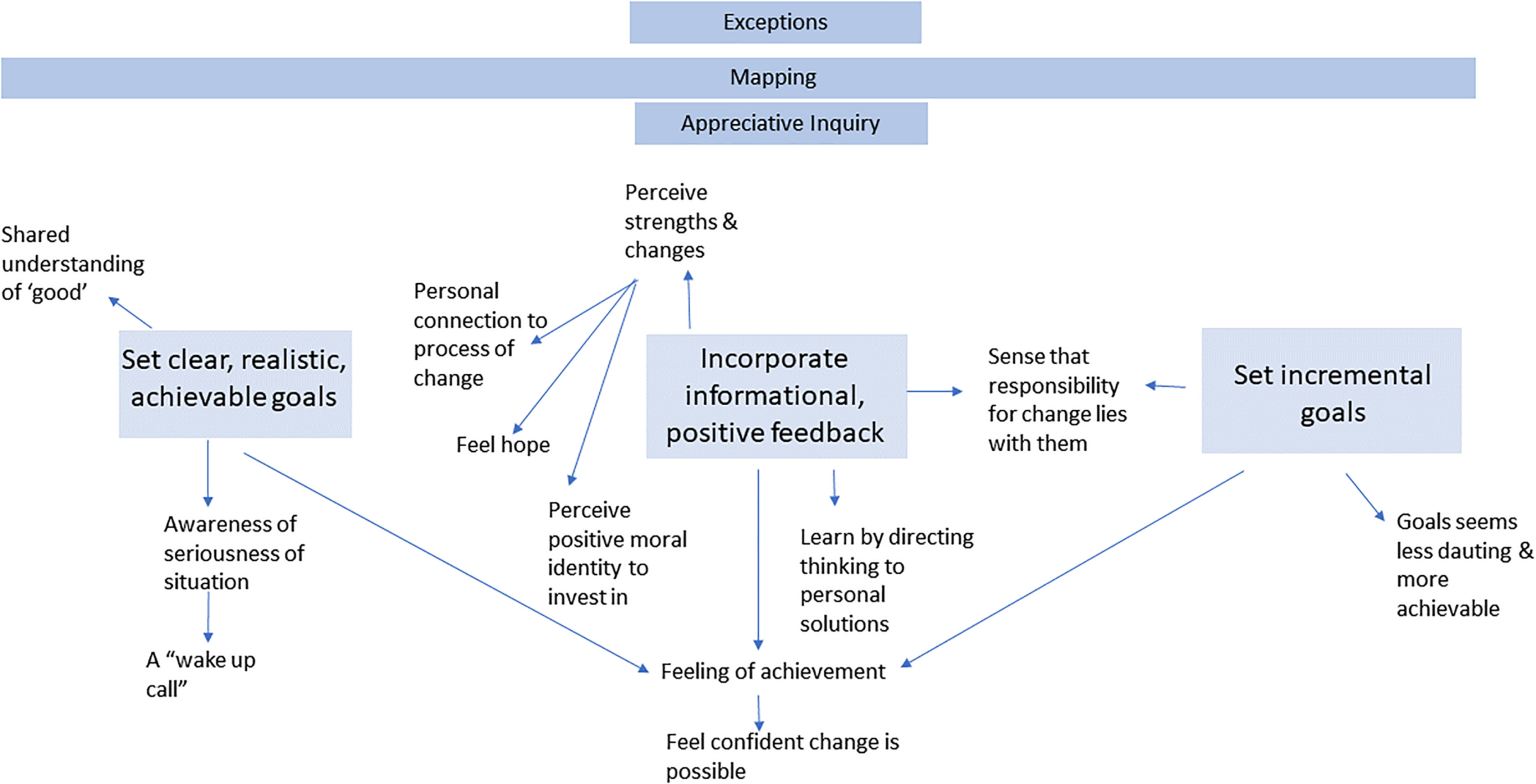
Competence supporting strategies and mechanisms

**FIGURE 5:**
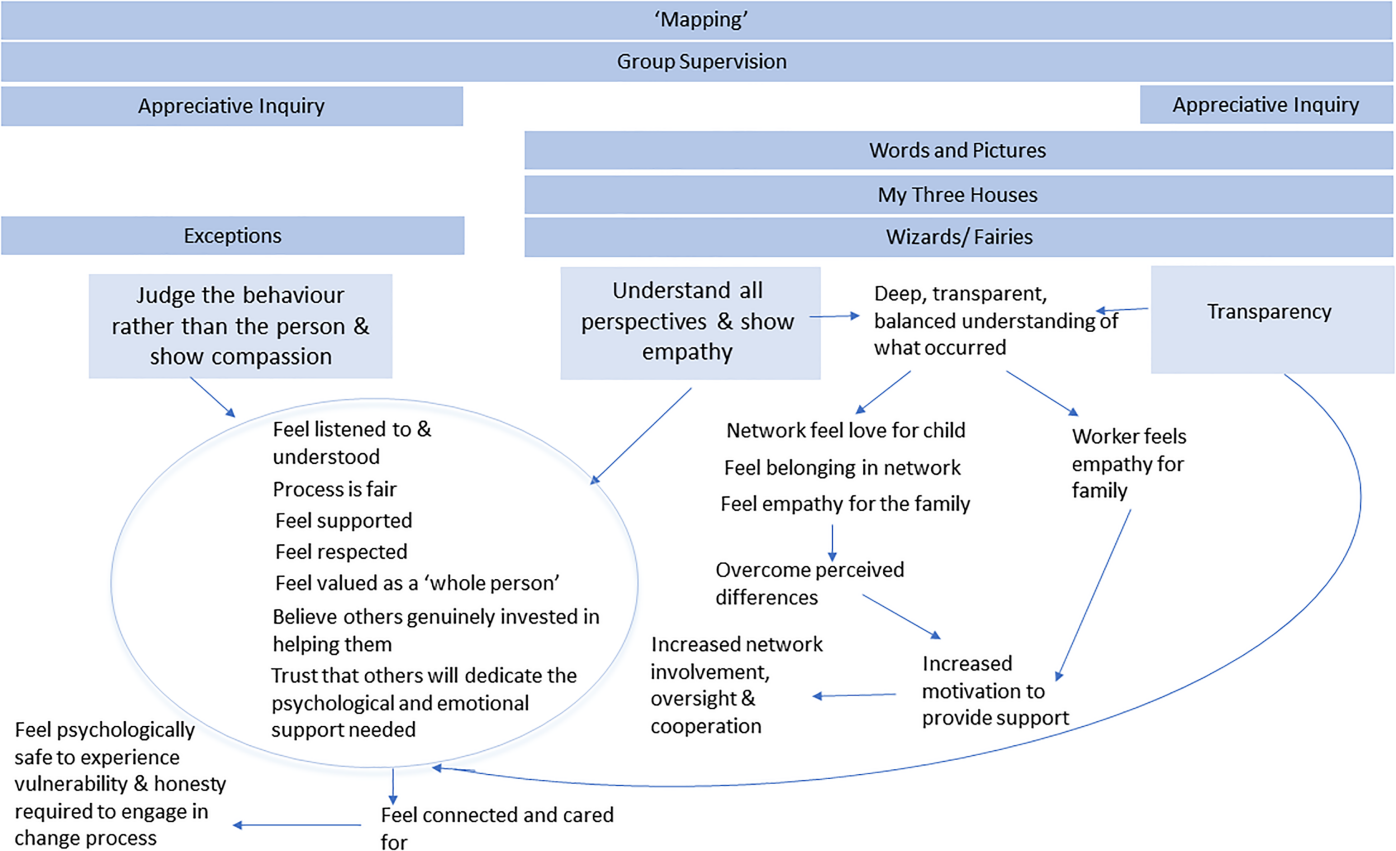
Relatedness supporting strategies and mechanisms

Throughout the following sections, each theorized mechanism of change is highlighted in bold and represented graphically in Figures 3, 4, 5. Tables 1, 2, 3 provide the sources in the SofS literature for each mechanism we refer to.

### 4.2 Autonomy supporting strategies

Empirical SDT research across diverse fields suggests that people are more likely to change and sustain their behaviour and experience well‐being when they experience, what SDT refers to as “autonomy support” (Ryan & Deci, 2017). Autonomy here is not the same as independence or freedom from external influence. Indeed, limit setting can be an important part of the process, but limits can be set in either controlling or autonomy‐supportive ways (Ryan & Deci, 2017). Rather, autonomy is about helping individuals recognize that they can make choices regarding their behaviours such that behaviours become self‐endorsed, feel in keeping with the person's own interests and values and are engaged in with a sense of volition (Ryan & Deci, 2017, p. 10).

SDT research has found that people are supported to experience ‘autonomy’ when they are (a) provided with opportunities for participation and choice and do not feel pressured or manipulated towards certain outcomes and (b) helped to formulate personally meaningful reasons for changing their behaviour (Ryan & Deci, 2017). In multiple other fields, compared with controlling approaches, these strategies are more likely to see clients develop a willingness to experiment, learn and grow and to maintain therapeutic gains (Ryan & Deci, 2017, pp. 442–444). By contrast, SDT research indicates that using controlled motivation techniques, including contingent rewards or power dynamics, can create an extrinsic focus that is likely to undermine people's willingness to be engaged and their propensity to engage in change behaviour (Ryan & Deci, 2017, p. 445). Figure 3 depicts the theory of how SofS strategies support autonomy, and Table 1 lists the literature referring each mechanism described in the narrative account that follows.

**TABLE 1:** Autonomy supporting strategies and mechanisms: supporting literature

Strategies: (a) enabling choice and participation, avoiding pressure and manipulation; (b) Personally meaningful reasons for change	
Mechanisms	Supporting literature
Feel understood	Skrypek et al., 2012; Turnell & Edwards, 1999; Munro et al., 2020; Bunn, 2013; Brent Council, 2017; Department of Child Protection, 2011
Reduces power differential	Reekers et al., 2018; Turnell & Edwards, 1999
Feel element of control	Nelson‐Dusek & Idzelis Rothe, 2015; Turnell & Edwards, 1999; Baginsky et al., 2017
Feel responsibility for change lies with them	Holmgård Sørensen, 2013; Baginsky et al., 2017; Turnell & Edwards, 1999
Feel ideas are valued	Keddell, 2011b; Sheehan et al., 2018; Turnell & Edwards, 1999
Feel trusted	Keddell, 2011a; Hayes et al., 2014; Holmgård Sørensen, 2013; Rothe et al., 2013
Feel respected rather than blamed	Beattie, 2013; Keddell, 2014; Turnell & Edwards, 1999; Hayes et al., 2014; Turnell et al., 2007
Learn solution finding	Keddell, 2011a
More clearly understand what needs to change	Skrypek et al., 2012; Holmgård Sørensen, 2013; Lohrbach & Sawyer, 2004; Turnell & Edwards, 1999; Hayes et al., 2014
Feel ownership and investment in process	Skrypek et al., 2012; Turnell & Edwards, 1999; Turnell & Murphy, 2017; Keddell, 2014; Caslor, 2011; Sheehan et al., 2018; Brent Council, 2017

#### 4.2.1 Enabling choice and participation and avoiding pressure and manipulation

In keeping with SDT, SofS's programme strategies hold that families will be more likely to engage in meaningful and sustainable behaviour change if they are provided with opportunities for participation and choice and if pressure and manipulation towards outcomes are avoided (Turnell & Edwards, 1999). In SofS, the mapping process (risk assessment) aims to enable choice and participation. Rather than telling families what to do, social workers make transparent their role, possible outcomes for the family and the child protection agency's ‘bottom line’ requirements for what they need to see to disengage. The social worker then engages the family, children and the network—‘every person naturally connected to the child’—in a questioning approach called ‘appreciative inquiry’ (AI), to elicit the family and network's ideas for how they can achieve the goals (Turnell & Edwards, 1999 p.33).

Participants acknowledged the challenges of ensuring families feel a genuine sense of choice and participation where engagement is often not voluntary and “bottom line” requirements need to be held to ensure child safety. Yet they reported that it is possible to create choice and participation. Choice and participation are effective strategies for behaviour change because they **reduce (not eliminate) the worker–family power differential**. This makes people feel **trusted, respected** rather than blamed and **understood** and that their **ideas are valued**. Choice and participation enable people to feel some **ownership** of the process, which can make them feel **invested** in it and **empower** them to feel capable of change.

Further, choice and participation, facilitated through AI and mapping, can help families to develop good judgement and creativity in solution finding. This supports long‐term behaviour change and thus sustainable family safety. This is because these strategies offer opportunities to personally **reflect** and **learn** through practicing solution finding, thus **integrating**, and **internalizing** new behaviour:

If staff [tell families what to do], families do not think through themselves what needs to happen, so they are not going to make changes that are going to be lasting.

[Participant A2: Architects group]

#### 4.2.2 Personally meaningful reasons for change

Directly in keeping with SDT, through ‘**mapping**’, SofS aims to find a ‘shared goal’ with the family that is focused on the family's own reasons for changing their behaviour. Determining a shared goal makes family members more likely to change their behaviour because it offers an opportunity for family members to more **clearly understand** what needs to change, **feel understood** and to feel that they have a **more equal**, less authoritative relationship with social workers. This can facilitate families to **feel invested in** and **value** the social work process. Moreover, creating a shared goal helps to **empower** families to make change because they will feel an element of **control** and **agency**. This offers an opportunity to internalize motivation since it implies that the **responsibility** for making change lies with them.

Participants acknowledged that it is not always possible to find a shared goal with families but reported that with a questioning and curious approach workers usually achieve this:

It is naive to think that you can bring out rules and tell people how to behave 'cause that's the opposite of autonomy for me. Where it does not work is where what people have been told to do does not matter to them. The difference with Signs of Safety is that […] it generally wants to work with people on what matters to the children, what matters to the family and then help them come up with ideas and solutions to make it happen. And I think for me, that's the driver that sets the model apart from anything else.

[Participant E: trainer and consultant group]

## 5 COMPETENCE SUPPORTING STRATEGIES

SDT research suggests that autonomously motivated behaviour change is facilitated through supporting a feeling of ‘competence’. People can be supported to feel competent when they are (a) helped to formulate clear, realistic, achievable goals; (b) are provided with positive, informational feedback regarding progress; and (c) encouraged to believe that they are capable of change through ‘optimal challenges’ conceived as ‘a more proximal goal, readily reached through smaller achievable steps’. These should be readily but not easily mastered and not be overly stressful or demanding (Ryan & Deci, 2017, p. 449). SDT suggests that supporting individuals to feel competent is important because to achieve outcomes and maintain their sense of well‐being, ‘people need to feel able to operate effectively within their important life contexts’ and that feeling of competence is easily undermined if challenges are too difficult, negative feedback is pervasive or person‐focused (Ryan & Deci, 2017, p. 11). Figure 4 details the specific SofS mechanisms, situating them within the SDT framework, and Table 2 outlines the references for each mechanism.

**TABLE 2:** Competency supporting strategies and mechanisms: supporting literature

Strategies: (a) clear, realistic, achievable goals; (b) Incorporating informational and positive feedback; (c) Setting incremental goals	
Mechanism	Supporting literature
Shared understanding of good	Lohrbach & Sawyer, 2004; Holmgård Sørensen, 2013; Turnell & Edwards, 1999; Munro et al., 2016 Keddell, 2011a; Hayes et al., 2014; Turnell & Murphy, 2017; Baginsky et al., 2017
Awareness of seriousness of the situation and ‘a wakeup call’	Gardner, 2008; Rothe et al., 2013 Lohrbach & Sawyer, 2004; Reekers et al., 2018; Skrypek et al., 2012; Holmgård Sørensen, 2013; Hayes et al., 2014; Turnell & Edwards, 1999; Bunn, 2013
Perceive strengths and changes	Turnell et al., 2007; Bunn, 2013; Reekers et al., 2018; Keddell, 2014; Caslor, 2011; Hayes et al., 2014; Salveron et al., 2015; Turnell & Edwards, 1999; Baginsky et al., 2017
Personal connection to the process of change	Keddell, 2011a; Skrypek et al., 2012; Bunn, 2013; Keddell, 2011b; Turnell & Edwards, 1999
Feel hope	Skrypek et al., 2012; Turnell & Edwards, 1999; Turnell & Murphy, 2017; Keddell, 2014; Hayes et al., 2014; Sheehan et al., 2018
Perceive positive moral identity	Keddell, 2014
Learn by directing thinking to personal solutions	Hayes et al., 2014; Reekers et al., 2018; Rothe et al., 2013; Stanley et al., 2018
Feel confident change is possible	Turnell & Edwards, 1999; Sheehan et al., 2018; Beattie, 2013; Baginsky et al., 2017; Turnell et al., 2007; Keddell, 2011a; Keddell, 2014
Sense that responsibility for change lies with them	Sheehan et al., 2018; Baginsky et al., 2017; Skrypek et al., 2012
Goals seem less daunting & more achievable	Turnell & Edwards, 1999
Feeling of achievement	Turnell & Edwards, 1999

### 5.1 Clear, realistic, achievable goals

In keeping with SDT, SofS seeks to specify clear, realistic, achievable goals. The ‘mapping’ process aims to make goals clear by specifying what needs to be done, rather than what families should stop doing. It aims to detail specific, measurable, observable and positive behaviours in jargon‐free language. Further, SofS emphasizes the importance of being honest, transparent and clear about the allegations and actions the agency might take (Turnell & Edwards, 1999, p.72).

The mapping process, by focusing on clear, behaviourally specific goals, offers an opportunity for workers and families to develop a **shared understanding** of what good work looks like. This can motivate behaviour change by providing a **feeling of achievement** for current positive behaviours. By supporting **awareness**, it may elicit a ‘**wake up call**’ for the family and wider network and for the social worker about the seriousness of the situation if worrying behaviours continue.

I think having safety goals that are very explicit and are achievable and are setting out what we want to see. Not ‘you must not’ but what we need to see is. […] even simple things like when we ask a scaling question, we always start with 10, what the best scenario would look like. So even those small things make a massive difference because we are building that hope and vision for the family. So we are suggesting that there's a possibility that you can do this.

[Participant B: trainer and consultant group]

### 5.2 Incorporating informational and positive feedback

SDT defines informational feedback as ‘non‐evaluative, it is about the behavior not the person’ (Ryan & Deci, 2017, p. 452) and SDT research has emphasized the importance of incorporating positive feedback and ensuring that all feedback is offered rather than imposed (Ryan & Deci, 2017, p. 452). Directly in line with this, a key feature of SofS is balanced, informational feedback that focuses on specific behaviours rather than the person (Turnell & Edwards, 1999). In SofS, this is achieved through the ‘mapping’ and ‘scaling’ process. These establish, not only ‘worries’ but also ‘what's working well’ to create a balanced assessment of ‘what needs to happen’. Positive, informational feedback is also incorporated through SofS's use of ‘appreciative inquiry’, which focuses on recognizing and celebrating behaviourally specific aspects of positive behaviour in families. Finally, positive, informational feedback is provided through identifying and understanding the reasons for ‘exceptions’. These are situations where families acted positively.

Participants strongly emphasized the importance of balance in the assessment process and that the focus on strengths should not be misunderstood to imply that ‘worries’ are not equally important. Rather the focus is on the *incorporation* of strengths in the assessment process because these are often missing from other approaches and their absence can mask viable possibilities to keep the child safe within the family network and avoid taking them into care.

Incorporating behaviourally specific positive feedback was seen to engage **motivation** by offering an opportunity for families to **believe that they are capable of change** because they **see strengths and changes**. This could create **hope** that they could do better and a sense of internalized **responsibility** that the ability to change lies with them. The idea of building on their own strengths can create **personal connection** to the process of change.

In addition, incorporating positive feedback can build family safety because it supports people to **learn**. This is because positive feedback helps people identify and build on what has already worked for them personally, thereby directing their **thinking to solutions** to problems. As the example below illustrates, this was seen to internalize and integrate motivation for change:

[Building on what has worked previously is important because] it has the assumption that there is already knowledge there. It does not come from outside. Someone has to teach me or tell me, do this, do that […] that builds a stronger foundation and engagement.

[Participant H: trainers and consultants group]

In the case of families, incorporating positive feedback is also motivating because it enables parents to embrace and invest in a **positive moral identity** of caring and competent parents, while acknowledging harm. Conversely, traditional child protection frameworks that describe parents only by the risk they pose, remove the opportunity for parents to occupy a positive moral identity and may thereby increase the chances that parents will react by becoming hostile and demotivated. Parents' sense of **hope** and **confidence** can be further reinforced by exploring ‘exceptions’ since these help situate harmful behaviour as situation specific, rather than pathological, thereby opening up a space in which the individual may feel **empowered** to identify what might help them to behave differently.

### 5.3 Setting incremental goals

SDT emphasizes the utility of smaller, proximal goals that can encourage people to believe that they can engage in the appropriate behaviours. Mirroring this, SofS processes establish intermediate, specific, smaller goals that incrementally build to larger ones (Turnell & Edwards, 1999, p.109–110). Setting intermediate goals was seen to build cooperation and motivation by supporting families to feel a sense of **confidence** in their own ability to make change, which facilitates the internal integration of motivation. Confidence can be built because intermediate goals give a sense of **achievement** and make goals seem **less daunting** and **more achievable** (Turnell & Edwards, 1999 p.43). Further, intermediate goals support safety by offering lower‐risk opportunities for staff to **assess** whether the family can make progress. This also supports social workers to feel **confident** in their assessment (including assessments to remove children) and, where the family achieve the intermediate goal, it **motivates** the social worker (as well as the family) to continue to offer support because they too feel a sense of **achievement**:

I think people think you have always got to present something amazing. ‘I stopped a child coming into care!’ Where it could just be ‘I had a phone call with mom and it's the first time she has not put the phone down on me.’ I think people can then sort of, you know, feel that it's OK for it to be a slight improvement. It does not have to be this ‘Oh, and I rescued these families.’

[Participant L: trainer and consultant group]

## 6 RELATEDNESS SUPPORTING STRATEGIES

SDT research has found that people tend to experience ‘relatedness’—feeling cared for or connected to others—when they perceive that significant others are genuinely invested in them and their well‐being, understand the difficulties they are facing and can be trusted to dedicate psychological and emotional resources to support them. SDT research therefore strongly suggests that the nature of the *relationship* between those on the supporting and receiving ends of change affects the likelihood of success. The research suggests that people are unlikely to internalize values or regulations from individuals they do not feel connected to or see as caring for them and that connectedness provides a sense of security that supports personal change. Therefore, SDT suggests that the ‘relatedness’ people experience is a vital contributory factor to ‘autonomous motivation’, which is conductive to sustainable behaviour change and well‐being (Ryan & Deci, 2017, pp. 444–447).

SDT research has demonstrated the importance of (a) engaging in a way that judges the behaviour rather than the person and treats them with compassion, interest, curiosity and a questioning approach that avoids blame; (b) understanding and empathizing with clients' perspectives as a means to invite and strategize a collaborative effort to overcome barriers to change; (c) transparency (Ryan & Deci, 2017, pp. 444–447).

In keeping with this, the SofS architects state that SofS places ‘relationships’ at the heart of the approach. The interaction between the worker and the service recipient is the ‘key vehicle for change’ (Turnell & Edwards, 1999 p.33). The specific SDT strategies are also mirrored in SofS. (A) As discussed above, SofS aims to judge the behaviour rather than the person, avoid blame, exhibit compassion by incorporating informational, balanced feedback into mapping and use a questioning through ‘appreciative inquiry’. (B) SofS emphasizes the importance of seeking to understand and acknowledge all family members' positions (Turnell & Edwards, 1999). It offers tools to achieve this including, ‘Words and Pictures’ and the child‐centred tools, ‘My Three Houses’ and ‘Wizards/Fairies’ as well as mapping. (C) Through the mapping process, Words and Pictures and My Three Houses, the approach aims to be transparent about the abuse and the allegations and the actions the agency might take and what it expects from families (Turnell & Edwards, 1999). As Figure 5 illustrates, the findings suggest that many of the same detailed mechanisms are at play across these three strategies. Therefore, below the three strategies are grouped into two overlapping categories, for which we detail the mechanisms. Table 3 outlines the references relating to each detailed mechanism.

**TABLE 3:** Relatedness supporting strategies and mechanisms: supporting literature

Strategies: (a) judging the behaviour and understanding all perspectives with empathy; (b) understanding perspectives and being transparent	
Mechanisms	Supporting literature
Feel listened to and understood	Keddell, 2011a; Keddell, 2011b; Lwin et al., 2014; Turnell et al., 2007; Baginsky et al., 2017; Brent Council, 2017; Hayes et al., 2014; Keddell, 2014; Munro et al., 2016; Nelson‐Dusek & Idzelis Rothe, 2015; Bunn, 2013; Lohrbach & Sawyer, 2004; Holmgård Sørensen, 2013; Turnell & Edwards, 1999; Munro et al., 2020; Caslor, 2011
Process is fair	Hayes et al., 2014; Turnell et al., 2007; Nelson‐Dusek & Idzelis Rothe, 2015; Keddell, 2014
Feel supported	Skrypek et al., 2012; Gardner, 2008; Reekers et al., 2018; Sheehan et al., 2018; Lwin et al., 2014; Hayes et al., 2014; Keddell, 2014; Turnell & Edwards, 1999
Feel respected/feel respect for	Lohrbach & Sawyer, 2004; Hayes et al., 2014; Beattie, 2013; Holmgård Sørensen, 2013; Nelson‐Dusek & Idzelis Rothe, 2015; Rothe et al., 2013; Skrypek et al., 2012; Turnell & Edwards, 1999; Keddell, 2014; Sheehan et al., 2018; Turnell & Murphy, 2017
Feel valued as a ‘whole person’	Salveron et al., 2015
Believe others are genuinely invested in helping them	Nelson‐Dusek & Idzelis Rothe, 2015; Skrypek et al., 2012; Turnell & Edwards, 1999; Munro et al., 2016
Trust others will dedicate the psychological and emotional support needed	Turnell & Edwards, 1999
Deep transparent balanced understanding of what has occurred	Rothe et al., 2013; Turnell & Edwards, 1999; Baginsky et al., 2017; Keddell, 2014; Bunn, 2013
Network have love for child, feel belonging to network. Network feel empathy for family	Keddell, 2011a; Keddell, 2011b; Skrypek et al., 2012; Rothe et al., 2013; Nelson‐Dusek & Idzelis Rothe, 2015
Worker feels empathy for the family	Brent Council, 2017; Skrypek et al., 2012,
Increase network involvement, oversight and cooperation	Holmgård Sørensen, 2013; Turnell & Edwards, 1999; Munro & Turnell, 2018; Turnell & Murphy, 2017
Feel connected and cared for	Skrypek et al., 2012; Turnell & Edwards, 1999
Feel psychologically safe	Keddell, 2014; Lwin et al., 2014; Reekers et al., 2018; Sheehan et al., 2018

### 6.1 Judging the behaviour and understanding all perspectives with empathy

The findings suggest that (a) judging the behaviour rather than the peson and (b) understanging all perspectives and showing empathy, offers the opportunity for families to integrate and internalize motivation to change their behaviour and to experience a greater sense of well‐being because they can come to **feel connected to and cared for** by those engaging them in the process. These strategies offer the opportunity for families to feel **listened to, understood, supported, respected** and that the process is **fair**. They feel valued as a ‘**whole person**’, who has strengths as well as problems and **believe that their social worker genuinely wants to help** them and that they will **dedicate the psychological and emotional support** needed to help them to change. Due to this, families feel **connected to** and **cared for** by those supporting them to change. This study's findings offer an extension of the current theory available in the literature to suggest that feeling cared for can support motivation to change because it allows families to overcome feelings of **anxiety** and **fear** such that they may feel **psychological safe** to experience the **vulnerability and honesty** required in a process of change. This may require families to engage with their social worker in difficult and painful conversations and often to confront shameful events:

When it comes to having no trust, what you are left with is a human being, either in fight, flight or freeze. And it's really hard to facilitate change happening when you have got people just fighting for their lives. So that takes away all the trust, the belief, the faith, and ultimately, they do not see that you are there to help them. You're out to get them. And I think the Signs of Safety processes can help. I do not think it magically makes it go away, but it definitely makes the process more open, more humble and what G was saying we are firm but fair and I think families can see it a little bit more that actually they are trying to do something good here.

[Participant E: Trainer & Consultant group]

### 6.2 Understanding perspectives and being transparent

This study's findings suggest that (a) understanding and empathizing with clients' perspectives and (b) being transparent can help to increase network motivation to provide support to the family, thereby offering an opportunity for greater child safety. As noted above, SDT research has found that ‘relatedness’, which underpins ‘autonomous motivation’ can be satisfied, not just by feeling cared for, but by feeling belonging and significance amongst others through experiencing oneself as giving or contributing to others (Ryan & Deci, 2017, p. 11). In keeping with this, the findings suggest that the SofS tools, including ‘Words and Pictures’ and ‘My Three Houses’ along with ‘appreciative inquiry’ and mapping, offer ‘relatedness supporting’ opportunities for social workers and the family network, that can help internalize and integrate, and thereby strengthen their motivation to support the family.

These strategies offer the opportunity to make children safer because **transparency** and **balanced understanding** can bring about sufficient feelings of **love** for the child and **belonging** within a family network such that family members who have been in conflict may become motivated to **overcome their differences** to provide increased network involvement, oversight and cooperation. Because child abuse is a ‘syndrome of secrecy’, this offers the opportunity to overcome isolation, increase connection and ultimately keep children safer.

Abuse starts with secrecy and is related to isolation and kids are safer when there's more people involved in their lives [..] So we have a mom who has a biological mom and adoptive mom and they hate each other. But she needs both moms to help her raise the kids, but they will not be in the room together. The process of Words and Pictures and other things helps the family relate to each other. Put the child in the middle and the child tends to be the hub that holds the families together, even when adults do not like each other.

[Participant D2: directors' group]

In the case of social workers, these tools can support ‘autonomous motivation’ because, by helping social workers develop a **deep, transparent and balanced understanding** of what has occurred, they offer an opportunity for **empathy** that can inspire heartfelt motivation. For example:

Not to focus on the tool, but those processes [Words & Pictures] helped me relate to that family like in a way that I cannot explain it, like it's hard to put words to it because it's like I felt so invested in working with them no matter what the outcome. I really felt that I was giving them a fair shake at creating the opportunity for them to build a plan for these kids and those kids did go home […]. It just changed the entire way that I looked at my relationships with families […]. If it had not been just for different circumstances of life, I could have been the person on the other side of that table, and who did I want facilitating those meetings for me?

[Participant D2: directors' group]

## 7 CONCLUSION

In this article, we report findings from a Realist Synthesis of SofS, identifying SDT as a theoretical framework for a key aspect of SofS. Using literature and focus group data, we syntheses and theoretically informed the explanation of what key SofS tools and processes are expected to achieve in the family–worker interaction and why. We did not aim to test hypotheses using the literature but rather to deepen the initial theory so that this theory can be tested in future empirical research. Our work builds on the realist synthesis of Sheehan et al. (2018), who reported that SofS can lead to positive engagement with families. Their programme theory suggested that the most commonly assumed mechanism for child safety is shared understanding of and responsibility for minimizing risk to children and that this requires parents to trust and collaborate with workers. Informed by realist (Shearn et al., 2017) and philosophy of science (Cartwright, 2020; Cartwright & Hardie, 2012) literature, this realist review substantiates Sheehan et al. 's(2018) work and, importantly, deepens and structures the analysis using a well‐established middle range theory, SDT.

We highlight for the first time that SofS strategies mirror those that SDT research has found facilitate ‘autonomous motivation’. We have conceptualized SofS strategies as aiming to satisfy families' need to feel ‘autonomy’ (engaging with some sense of willingness) ‘competence’ (feeling effective) and ‘relatedness’ (feeling cared for) in the child protection process. Using SDT we assert that SofS aims to support families to experience ‘autonomous’ rather than ‘controlled’ motivation and expects that this is more likely to promote and sustain family behaviour change and well‐being. This hypothesis is supported by SDT research across diverse fields but has not, to the best of our knowledge, been tested in a child protection social work context and we recommend empirical testing.

Fundamentally, our review offers a practical contribution to social work. Previous research has found that SofS is often not implemented as intended and there can be confusion about what SofS is (Baginsky et al., 2017; Baginsky et al., 2021; Roberts et al., 2019). The theory of SDT alongside the SofS‐specific mechanisms we synthesize, can inform SofS training, practice and implementation because we offer a deeper explanation of why SofS strategies are important and more precisely specify what each aims to achieve. Our findings emphasize the importance of social workers avoiding a focus simply on the tools of SofS. It would be possible, for example, to use SofS tools in a controlling rather than autonomy supporting way, though this would not constitute SofS. Rather, while tools are important, SDT implies that SofS can be explained as an ‘interpersonal style’ (Markland et al., 2005, p. 825), with outcomes highly influenced by how families feel in interactions with workers and, at an overarching level, whether families feel that interactions are autonomy supportive or controlling. Our findings emphasize that engaging in an autonomy supportive way in no way negates the importance of setting and enforcing ‘bottom lines’. Rather, via SDT, we show how SofS can be conceptualized as aiming to set and enforced limits in ways that support basic needs for autonomy, competence and relatedness, avoiding a controlling approach.

Our theoretical development offers the opportunity to empirically investigate whether families' perception of workers' behaviours as autonomy supportive or relatively controlling mediates outcomes in varying contexts, alongside safety measures e.g. setting and maintaining bottom lines. By this means, the findings offer a new avenue for evaluation research on SofS. Moving beyond checking whether SofS processes and tools have been used to explore whether those tools and processes can trigger their intended mechanisms and in what contexts this occurs, would help to fill in the ‘black box’ between SofS activities and outcomes. Future research could use a realist evaluation (Pawson & Tilley, 1997) to achieve this.

Finally, these findings are relevant beyond SofS because SofS is not the only practice framework that can be conceptualized as aiming to support ‘autonomous’ over ‘controlled’ motivation. We identify the absence of SDT‐informed research in social work as a significant gap in the literature and encourage empirical research to explore whether ‘autonomous motivation’ through ‘autonomy’, ‘competence’ and ‘relatedness’ support generally mediates the social worker–family relationship and the staff‐organizational relationship.

## 8 STUDY LIMITATONS

We note several study limitations. Firstly, this paper reports on the mechanisms underlying the worker–family relationship in the child protection process. As a Realist Synthesis, the study further investigated how mechanisms are triggered in contexts with varying outcomes and this is the reported elsewhere (Caffrey & Browne, n.d.). Our study also demonstrated how, in a ‘parallel process’ (Turnell & Murphy, 2017), at the organizational level, SofS can be conceptualized as aiming to support workers' basic human needs with the aim of supporting staff to experience ‘autonomous motivation’ and so feel motivated to implement SofS. However, presentation of these findings was beyond this paper's scope. Further, the theory we have proposed is inherently partial and open to progress and refinement (Pawson, 2013). Our study did not explore all the available literature on SofS and further incorporation could add to our findings. We also emphasize that our findings relate to a key aspect of SofS but do not constitute an explanation of SofS in its entirety. Future work could, for example, develop the theoretical underpinning for child engagement or balanced mapping.

## CONFLICT OF INTEREST

This work was supported by funding from Resolutions Consultancy.

## Data Availability

In relation to the review of literature: data sharing not applicable—no new data generated as no new data were created or analyzed in this part of the study. In relation to focus group data: due to ethical constraints, the author elects to not share data.

## ENDNOTE

^1^
Elia is an international not‐for‐profit organization comprising Elia's staff and SofS trainers and consultants. Further information available online (https://www.elia.ngo/about). The list of publication was compiled by Mike Caslor, Director of Research at Elia.

## APPENDIX A

List of signs of safety expert participants:

Mike Caslor, Sabien De Klerck, Beverley Edwards, Ann Gardeström, Caara Goodard, Denis Gorgon, Rosina Harvey‐Keeping, Ai Hishikawa, Deirdre Mahon, Haley Muir, Catherine Mullin, Eileen Munro, Terry Murphy, Nova Nilsdotter, Jo Ratcliffe, Stacey Steinbach, Leigh Taylor, Andrew Turnell, Penelope Turnell, Marieke Vogel, Joke Wiggerink, Pippa Young.
